# A cationic near infrared fluorescent agent and ethyl-cinnamate tissue clearing protocol for vascular staining and imaging

**DOI:** 10.1038/s41598-018-36741-1

**Published:** 2019-01-24

**Authors:** Jiaguo Huang, Cinzia Brenna, Arif ul Maula Khan, Cristina Daniele, Rüdiger Rudolf, Vincent Heuveline, Norbert Gretz

**Affiliations:** 1 Medical Research Center, Medical Faculty Mannheim, University of Heidelberg, Theodor-Kutzer-Ufer 1-3, 68167 Mannheim, Germany; 2 Institute for Medical Technology, University of Heidelberg and University of Applied Sciences, Theodor-Kutzer-Ufer 1-3, 68167 Mannheim, Germany; 3 Institute of Molecular and Cell Biology, Mannheim University of Applied Sciences, 68163 Mannheim, Germany; 4 Director of the Computing Centre, Heidelberg University, Im Neuenheimer Feld 293, 69120 Heidelberg, Germany

## Abstract

Understanding vascular structures and dysfunction is a fundamental challenge. This task has been approached by using traditional methodologies such as microscopic computed tomography and magnetic resonance imaging. Both techniques are not only expensive but also time-consuming. Here, we present a new method for visualizing vascular structures in different organs in an efficient manner. A cationic near infrared (NIR) fluorescent dye was developed with attractive features to specifically stain blood vessels. Furthermore, we refined the process of organ staining and harvesting by retrograde perfusion and optimized the subsequent dehydration and clearing process by the use of an automatic tissue processor and a non-toxic substance, ethyl-cinnamate. Using this approach, the time interval between organ harvesting and microscopic analysis can be reduced from day(s) or weeks to 4 hours. Finally, we have demonstrated that the new NIR fluorescent agent in combination with confocal or light-sheet microscopy can be efficiently used for visualization of vascular structures, such as the blood vessels in different organs e.g. glomeruli in kidneys, with an extremely high resolution. Our approach facilitates the development of automatic image processing and the quantitative analysis to study vascular and kidney diseases.

## Introduction

Changes in vascularization and vascular dysfunction have been associated with many diseases, including cardiovascular and kidney disease, making this an area of intense interest^1^. For example, the changes in the number and size of glomeruli (a tuft of capillaries) have been associated with a number of kidney and systemic diseases^2,3^. Brenner *et al*. hypothesized that a reduced number of nephrons increases the risk of hypertension and thereby is a risk factor for the development of chronic kidney disease^4^. Given the importance of glomeruli in kidney function, researchers need access to advanced techniques that are accurate and efficient for visualizing the structure of glomeruli. Our previous studies and other recent work show that using magnetic resonance imaging (MRI) allows a direct assessment of the number of glomeruli or computed tomography (CT) predicts acute coronary symptoms^5–7^. In these studies, cationic ferritin was used to accumulate in glomeruli and to stain the glomerular basement membranes through electrostatic binding with glycosaminoglycans (GAGs) and proteoglycan^3,5^, because GAGs are highly negatively charged molecules and expressed throughout the body either on the cell surface or the extracellular matrices^3,5^. The majority of GAGs are found in the body as proteoglycan components and are abundant in vessels, basement membranes of glomeruli in kidneys, cartilage and bone^8^. However, this MRI technique requires instrumentation (i.e. not easily available), expensive contrast agents, and suffers from relatively poor resolution that limits distinction between capillary tuft and Bowman’s capsule^9^. Therefore, there is a high demand to develop a superior technique for imaging vascular structures and visualizing the progressive stages of the related diseases in experimental animal models.

Due to its good spatial resolution, optical imaging in combination with the fluorescence staining of intracellular biomolecules is a widely used method to obtain single-cell resolution information in an organ or a whole body. Various dyes such as Alcian Blue, synthetic chromophores^10^ and charged green fluorescent protein^11^ have been used for the visualization of GAGs. However, the wavelength of such dyes is in the blue and green region of the spectrum. This fact limits their application in visualising and imaging the inner organ structures because of the poor penetration depth and the disturbance of the signal by auto-fluorescence of the tissue, compared to the red region of the spectrum. Although the vessels and glomeruli can also be visualized by staining endothelial cells with Alexa Fluor 647 labelled anti-CD31, this method results in an underestimation of glomerular number^12^. Therefore, new fluorescent agents with a suitable signal-to-noise ratio are needed for a specific staining and imaging of vascular structures in different organs.

Another obstacle in optical imaging of vascular structures of whole organs is tissue opacity^13,14^. This is due to scattering and absorption of light by lipids, organelles, and large protein clusters in the tissue^15^. Thus, tissue clearing is critical for improved optical imaging, especially of imaging whole organs. Although tissue clearing has been introduced more than a century ago, recently developed tissue clearing methods are most well-suited for efficient optical imaging^16–20^. Modern solvent based tissue clearing methods can generally be divided into two types: (i) aqueous solution based tissue clearing (e.g., Scale^21,22^, 3DISCO^23^, iDISCO^24^, SeeDB^25^, ClearT^26–28^, 2, 2′-thiodiethanol^29^) or (ii) organic solvents (e.g. benzyl alcohol-methyl salicylate^16^, benzyl alcohol–benzyl benzoate (BABB)^30^, dichloromethane^23^, and dibenzyl ether^31^). The aqueous solution based methods can result in highly transparent samples by removing lipids and homogenizing refractive index (RI) of the tissue, however, a long time (ranging from days to weeks) is required for completely clearing a whole organ. In contrast, organic solvents based tissue clearing can achieve clearing in a relatively short time period. However, most clearing solvents in use rely on toxic chemicals, for example, BABB, dichloromethane, and methyl salicylate, which are potent carcinogens^24^. Even worse is the concern about the drastic loss of fluorescence signal from staining during tissue clearing^18^. Thus, it is imperative to develop safer and more efficient tissue clearing protocols to protect researchers from exposure to harmful environment.

In the present work, we have proven that a new cationic near infrared (NIR) fluorescent dye specifically stains blood vessels. Furthermore, we accelerated the ethyl-cinnamate clearing process significantly. This approach combined with confocal or light-sheet microscopy allows a rapid visualization of vascular structures in different organs e.g. in kidney or heart.

## Results

### Design, synthesis and characterization of a NIR dye for staining vascular structures

One of the major shortcomings of using aforementioned fluorescent molecules in the blue and green regions of the spectrum is the low signal-to-noise ratio, which significantly compromises the accuracy of visualizing vessels^12^. Therefore, we developed a NIR fluorescent dye specifically staining vascular structures and exhibiting a deep penetration depth and a low fluorescent background. To design this dye, we have taken the unique structure of vessels and the glomerular capillary wall into consideration. Firstly, negatively charged GAGs are essential components in the endothelium of vessel walls^32^. Secondly, the glomerular capillary wall includes an endothelium with a fenestration (70–90 nm), a basement membrane (2–8 nm) and an epithelium with filtration slit embedded into podocyte extensions (4–11 nm)^32^. Owing to the combined effects of each layer of the glomerular capillary wall, macromolecules or particles with a hydrodynamic diameter (HD) of less than 6 nm can be filtered easily^32^. Apart from the size, the molecular weight threshold of glomerular filtration is thought to be 50 kDa^33^. Therefore, it has been postulated that macromolecules with a molecular weight exceeding this value cannot pass through the glomerular filter into urine under normal conditions^34^. In addition, the surface charge of macromolecules or particles also plays a key role in glomerular filtration because of the negatively charged GAGs. Therefore, we developed a fluorescent dye (MHI148-PEI) consisting of two key functional components: branched polyethylenimine (PEI) and a NIR heptamethine cyanine dye (MHI148). MHI148-PEI exhibits several attractive features: (i) the NIR wavelength provides a deep penetration depth and minimizes the interference of auto-fluorescence with biological tissues; (ii) the large molecular weight (50–100 kDa) prevents it from passing through the glomeruli into tubules; (iii) the positive charges derived from numerous cationic amino groups leads to its electrostatic attachment to GAGs and proteoglycans in vessels as well as the glomerular basement membrane.

**Figure 1:**
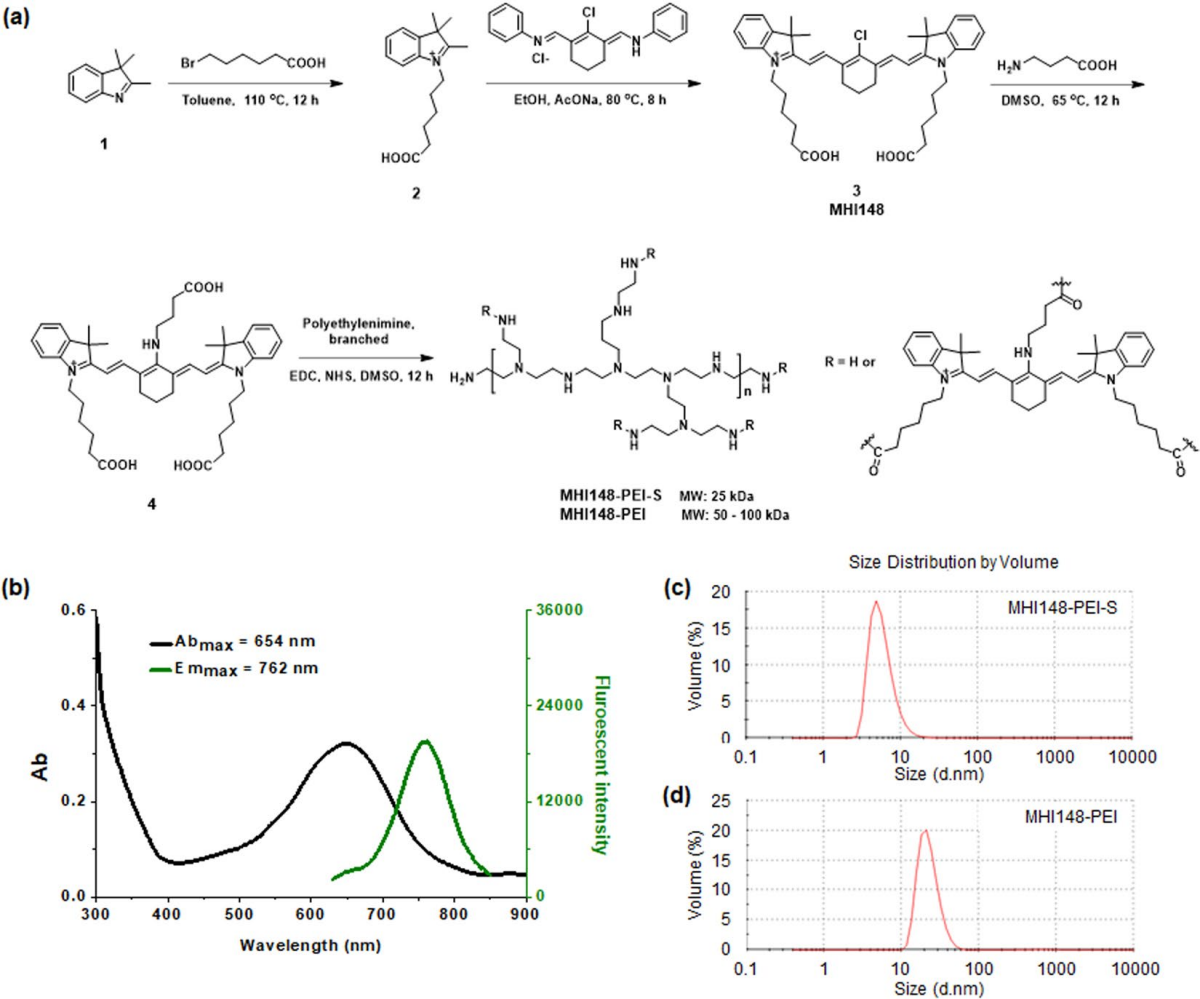
Overview of chemical structures and optical properties of MHI148-PEI. (**a**) Synthetic route of MHI148-PEI and MHI148-PEI-S. (**b**) The absorption and fluorescent emission spectra of MHI148-PEI in phosphate buffered saline (PBS) solution, Ab_max_: 654 nm, Em_max_: 762 nm. (**c**) and (**d**) The size distribution of MHI148-PEI-S and MHI148-PEI.

The synthetic route of the NIR fluorescent agent MHI148-PEI is illustrated in Fig. 1a. MHI148-PEI displays an absorption peak at 654 nm and an emission peak at 762 nm with a large Stokes shift of 108 nm (Fig. 1b). The absorption and emission spectra of MHI148 and compound 4 are depicted in Supplementary Fig. S2. This supercharged NIR dye is expected to stain vessels via electrostatic attachment. To test the design hypothesis, we used another branched PEI with lower molecular weight (25 kDa) to construct a second NIR agent (MHI148-PEI-S) and compared its molecular size and staining features on glomeruli in kidneys with that of MHI148-PEI. In dynamic light scattering (DLS) experiments, we found that the mean diameter in size distribution is 5 nm for MHI148-PEI-S (Fig. 1c). On the other hand, MHI148-PEI has a larger hydrodynamic diameter with a mean diameter over 10 nm (Fig. 1d), which is larger than the filtration pore threshold of the kidney (6 nm). Thus, these two cationic NIR dyes with different molecular size are expected to display different imaging features.

### Tissue dehydration and clearing with ethyl-cinnamate (ECi)

**Figure 2:**
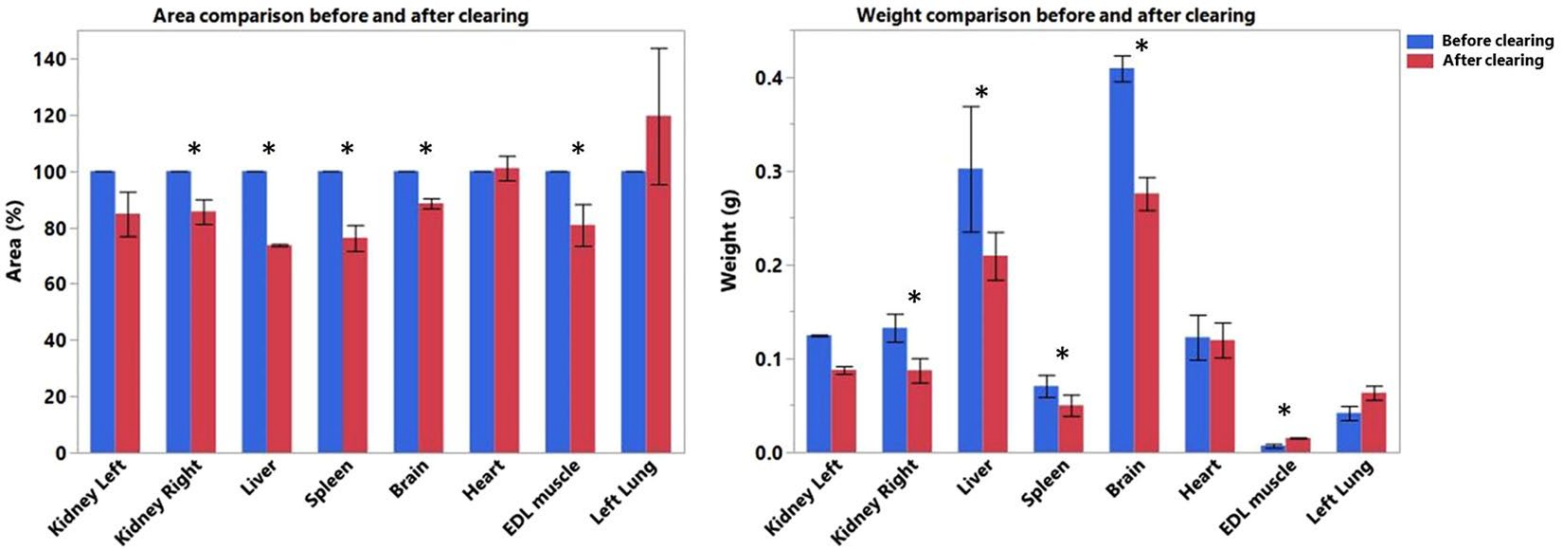
Shrinkage analysis of different organs based on weight (manual) and 2D surface area (automatic). Bar plot showing the weight (on right) and the area (on left) of the organs before and after clearing. The values for area are given as a percentage normalized to fixed percentage (100%) for organs before clearing. The area calculation was based on automatic image processing. The weight values are expressed in grams. Asterisks denote significance (p < 0.05) in a paired t test before and after clearing, error bars denote SD. The sample size is n = 3.

Klingberg *et al*. recently developed a new solvent based clearing method using ethyl-3-phenylprop-2-enoate (ECi)^12^. ECi is considered nontoxic according to the European directive 67/548/EWG and is a Food and Drug Administration (FDA) approved food flavor and additive for cosmetic products^35^. ECi-based tissue clearing comprises two steps: (i) dehydration using ethanol; (ii) lipid extraction and clearing by matching RI to the remaining dehydrated tissue’s index. Despite ECi is demonstrated to be an excellent clearing reagent for mammalian tissues, the procedure used in the previous report is cumbersome and time-consuming (roughly 16 to 20 hours)^12^. In contrast, our protocol lasts only 4 hours. After perfusion, the following organs were collected: right and left kidney, liver, spleen, left lung, heart, left lateral lobe of the liver, brain, extensor digitorum longus (EDL) muscle (for details see Material and Methods). The shrinkage of those organs was estimated by calculating the difference of weight and 2D surface of the organs (Fig. 2). A variable weight and surface change was observed in the cleared organs. A decrease in surface area of brain, kidneys, liver, spleen and EDL muscle (brain −11.5%, right kidney −14.4%, left kidney −15.2%, liver −26.3%, spleen −23.7%, EDL muscle −19.2%) and an increase of lung and heart (lung +19.2%, heart +1.1%) was noticed. Concerning the weight, we observed a gain for brain, kidneys, liver, spleen and heart (brain +0.133 g, right kidney +0.045 g, left kidney +0.037 g, liver +0.092 g, +spleen 0.021 g, heart +0.003 g) and a loss for EDL muscle and lung (EDL muscle −0.008 g, lung −0.021 g).

### Imaging and visualizing vascular structures in different cleared organs

**Figure 3:**
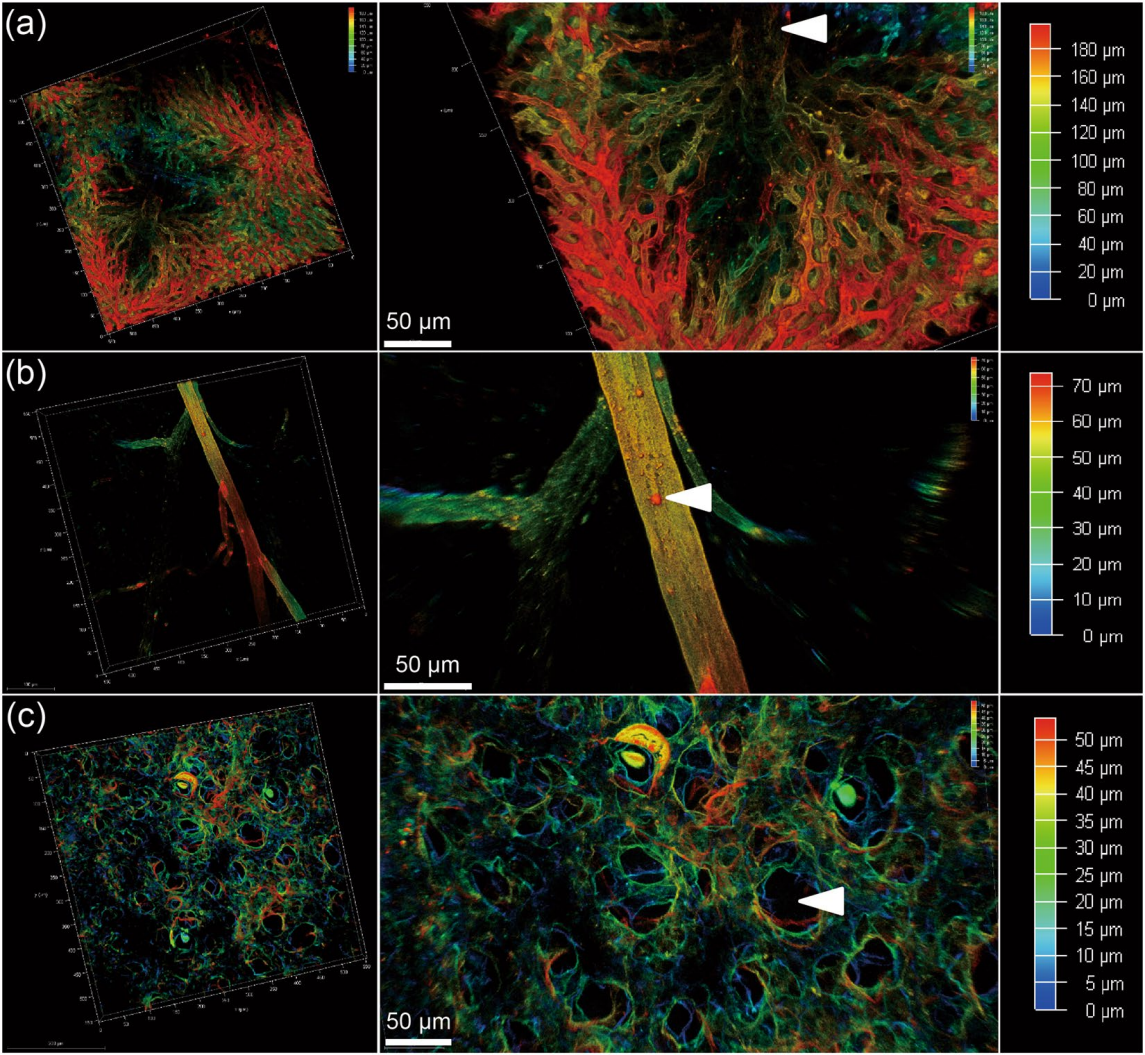
Sections of liver, skin and lung of a mouse are imaged using a 20x objective of a confocal microscope, after perfusion with MHI148-PEI and clearing with ECi. (**a**) Thin section from liver in 3D (left). Zoomed-in view shows central vein indicated by an arrowhead and capillary vessel network in true color (middle). A scanned depth of 180 μm was used as indicated by the depth bar with color coding (right). (**b**) Thin skin section from upper back of a mouse in 3D (left). Zoomed-in view shows a vessel with some branches (middle). A thin section of depth about 70 μm was scanned as shown in the depth bar with color coding (right). (**c**) Thin section from lung (left). Zoomed-in view shows alveoli as indicated by an arrowhead (middle). The depth bar with color coding is on the right hand side. The colored data in 3D are the structures and the black color is used for the background in each subfigure.

**Figure 4:**
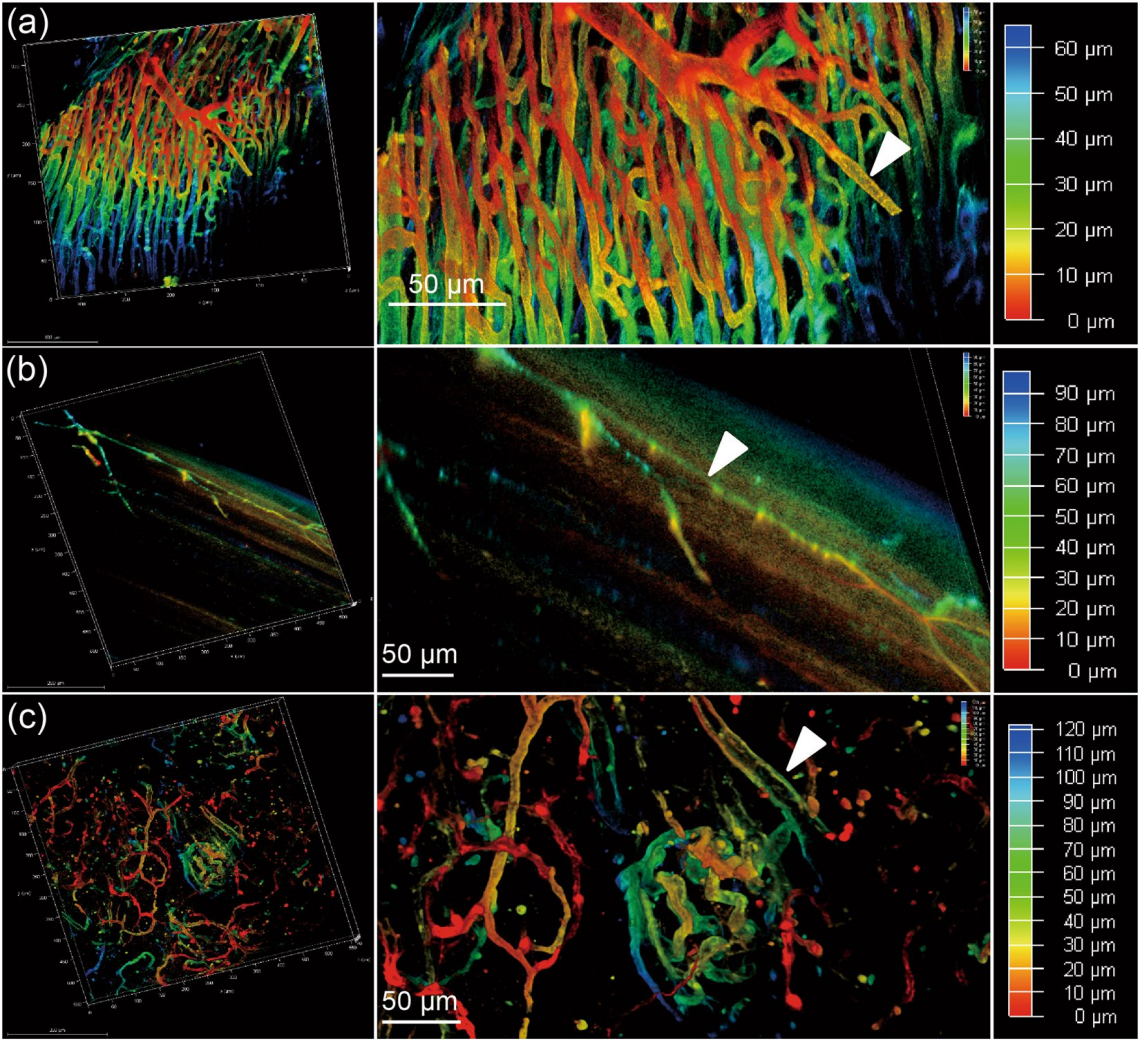
Sections of heart, muscle and pancreas of a mouse are imaged using a 20x objective of a confocal microscope, after perfusion with MHI148-PEI and clearing with ECi. (**a**) Thin section from heart in 3D (left). Zoomed-in view shows arteries fully stained by MHI148-PEI. A scanned depth of 65 μm was used as indicated by the depth bar with color coding (right). (**b**) Thin muscle section from the leg of a mouse in 3D (left). Zoomed-in view shows capillary vessels with some branches (middle). A thin section of depth of about 90 μm was scanned as shown in the depth bar with color coding (right). (**c**) Thin section from pancreas (left). Zoomed-in view shows vessels in pancreas. A thin section of depth of about 120 μm was scanned as shown in the depth bar with color coding (right). The colored data in 3D are the structures and the black color is used for the background in each subfigure.

**Figure 5:**
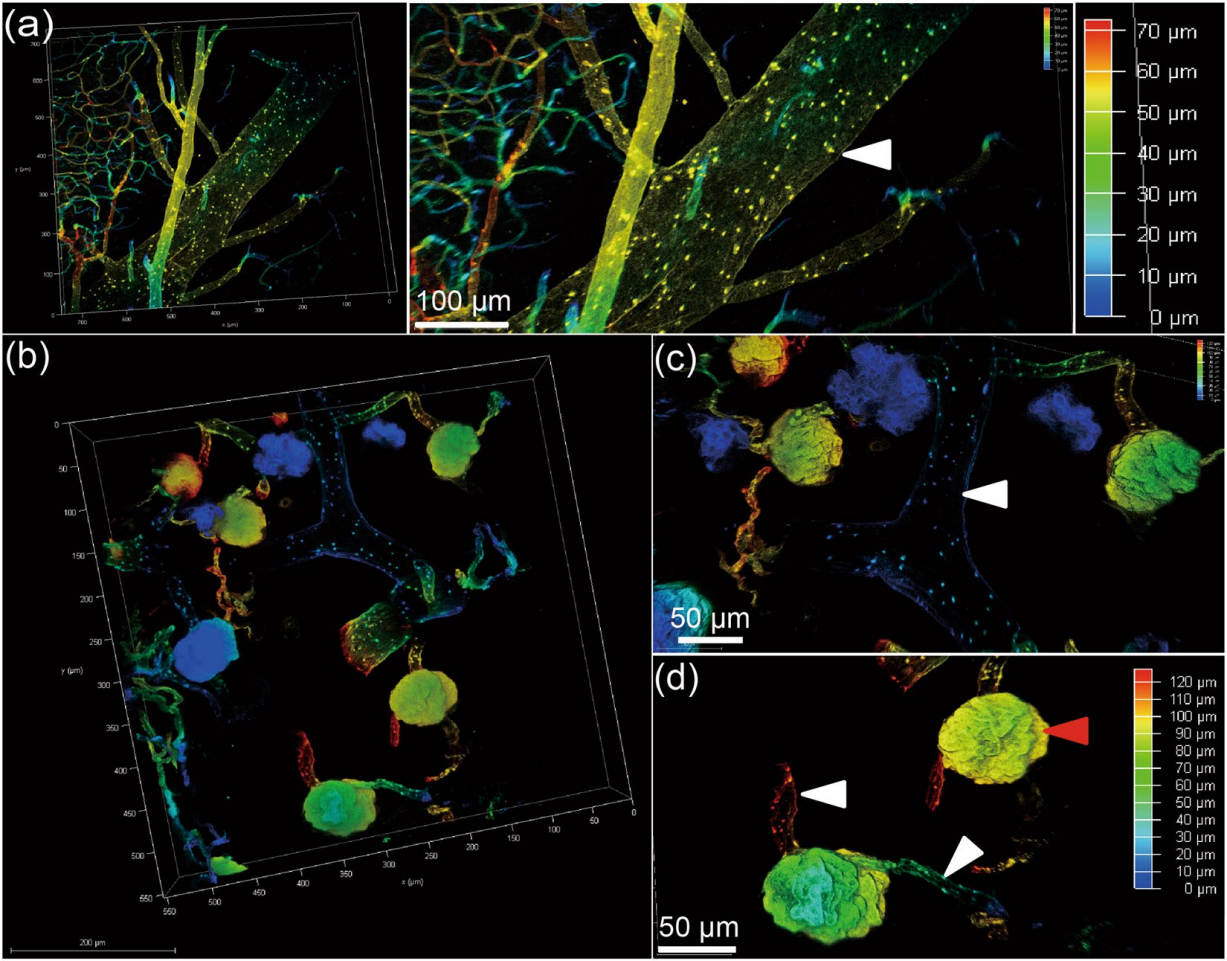
Sections of brain and kidney of a mouse are imaged using a 20x objective of a confocal microscope, after perfusion with MHI148-PEI and clearing with ECi. (**a**) Thin section from brain (left). Zoomed-in view shows vessels in brain. A thin section of depth about 70 μm was scanned as shown in the depth bar with color coding (right). (**b**) A thin section (white scale bar is 200 micro-meter) from left kidney of a mouse showing vessels and glomeruli in color against a black background. (**c**) Magnified view of (**b**) showing stained boundaries of a vessel in the middle with branches as indicated by the white arrowhead, and (**d**) further magnified view of (**b**) showing complete staining of glomerular basement membrane indicated by a red arrowhead. The efferent and afferent vessels are indicated by white arrowheads. The colored data in 3D are the structures and black color is used for the background in each subfigure.

To visualize vascular structures in different organs, we scanned the cleared tissues after MHI148-PEI staining by using confocal microscopy (Leica SP8). As shown in Figs 3a and 4a,c, the NIR window clearly visualized the vascular network and higher-order branches of blood vessels in cleared liver, heart and pancreas, suggesting the dye is still visible after the clearing process. Furthermore, to investigate whether the small vessels or capillaries can also be visualized, cleared skin and muscle with MHI148-PEI staining were imaged. Figs 3b and 4b show that the distribution of capillary vessels in skin and muscle can be clearly observed, which will allow precise quantitative evaluation of blood capillary size, length and branching. Figure 3c shows images of the alveolar wall in lungs. The basement membrane and extracellular matrix on the alveolar wall are composed of 3D fibrous mesh structures and contain various interconnected and intercalated macromolecules like GAGs^36^. Importantly, perfusion with MHI148-PEI can reveal the size difference in vascular architecture at a high resolution in the mouse brain (Fig. 5a). These results demonstrate that this cationic dye and ECi optical tissue clearing protocol facilitates vascular staining and imaging.

### Imaging and visualizing vessels and glomeruli in cleared kidneys

Next, we analyzed whether MHI148-PEI can also be used to visualize the structures of glomeruli in kidneys and found that this protocol is suited to visualize glomerular tufts and the vascular network with a high signal-to-noise ratio (Fig. 5b–d). Image stacks displayed all glomeruli and vessels as bright fluorescent areas in one three-dimensional stack (Fig. 5b). The high signal-to-noise ratio of the MHI148-PEI fluorescence signals will facilitate quantifying number and size of glomeruli.

**Figure 6:**
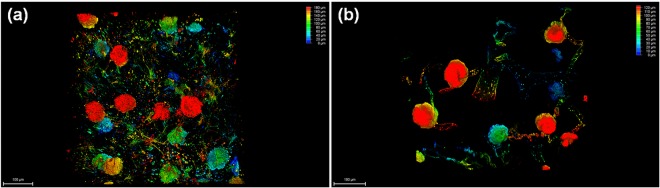
Sections of two kidneys from two mice are imaged using a 20x objective of a confocal microscope, after perfusion with MHI148-PEI and MHI148-PEI-S, respectively, and clearing with ECi. (**a**) A thin section from left kidney of a mouse showing vessels and glomeruli with MHI148-PEI-S staining. (**b**) A thin section from left kidney of a mouse showing vessels and glomeruli with MHI148-PEI staining.

In order to investigate whether the size of cationic NIR agents can affect the staining and imaging of glomeruli, both MHI148-PEI and MHI148-PEI-S were perfused in mice for kidney staining. We observed that MHI148-PEI remains within vessels and glomeruli without any filtration into tubules after perfusion, giving an extremely high signal-to-noise ratio (Fig. 6b). Conversely, a high background was observed in a cleared kidney after perfusion with MHI148-PEI-S. Very likely, this differential behavior of the dyes was due to the large molecular weight (50–100 kDa) and larger size (more than 10 nm) of MHI148-PEI on the one hand that prevents it from passing through the glomerular capillary wall into tubules. In contrast, the molecule size of MHI148-PEI-S was likely smaller than the threshold of glomerular filtration, leading to a leakage into the tubules. In summary, this demonstrates that MHI148-PEI outperforms MHI148-PEI-S, at least in the context of glomerular staining.

**Figure 7:**
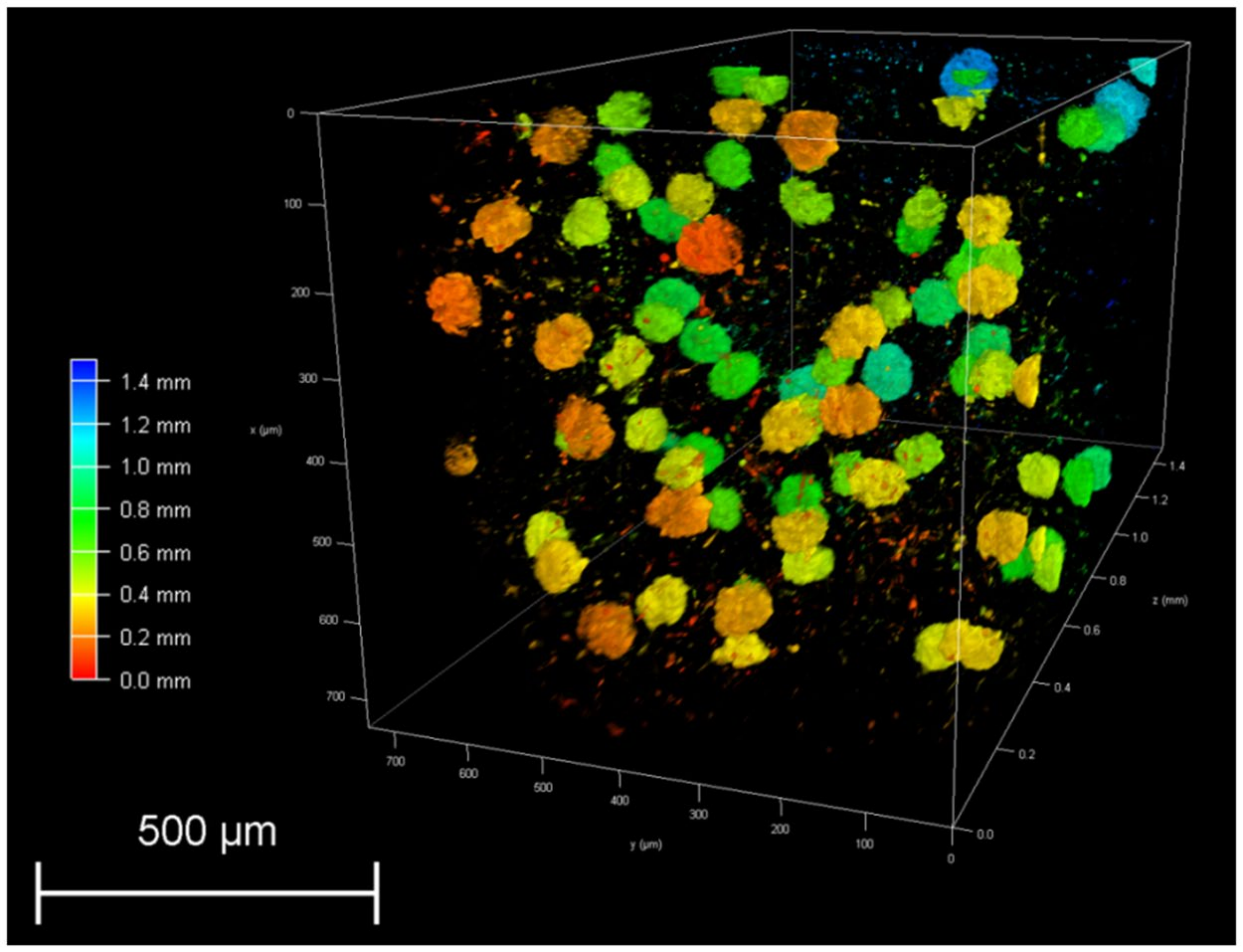
Visualization of 3D glomeruli in a portion of an ECi-cleared kidney scanned by light-sheet microscopy. A section of depth about 1.5 millimeters was scanned as shown in depth bar (left). Glomeruli are fully imaged after staining with MHI148-PEI. The size and number can be counted by using image processing and data analysis.

ECi-cleared whole organs can also be scanned by light-sheet microscopy (Fig. 7). Image stacks of a portion of one cleared kidney revealed that glomeruli with MHI148-PEI staining displayed bright fluorescence signals, which will also allow the quantification of the volume or size of all glomerular tufts in future studies. Thus, this comprehensive protocol can be used to investigate the vascular structures in different organs, and can serve as an efficient tool in the study of glomerular disease and other kidney diseases.

### Images analysis

**Figure 8:**
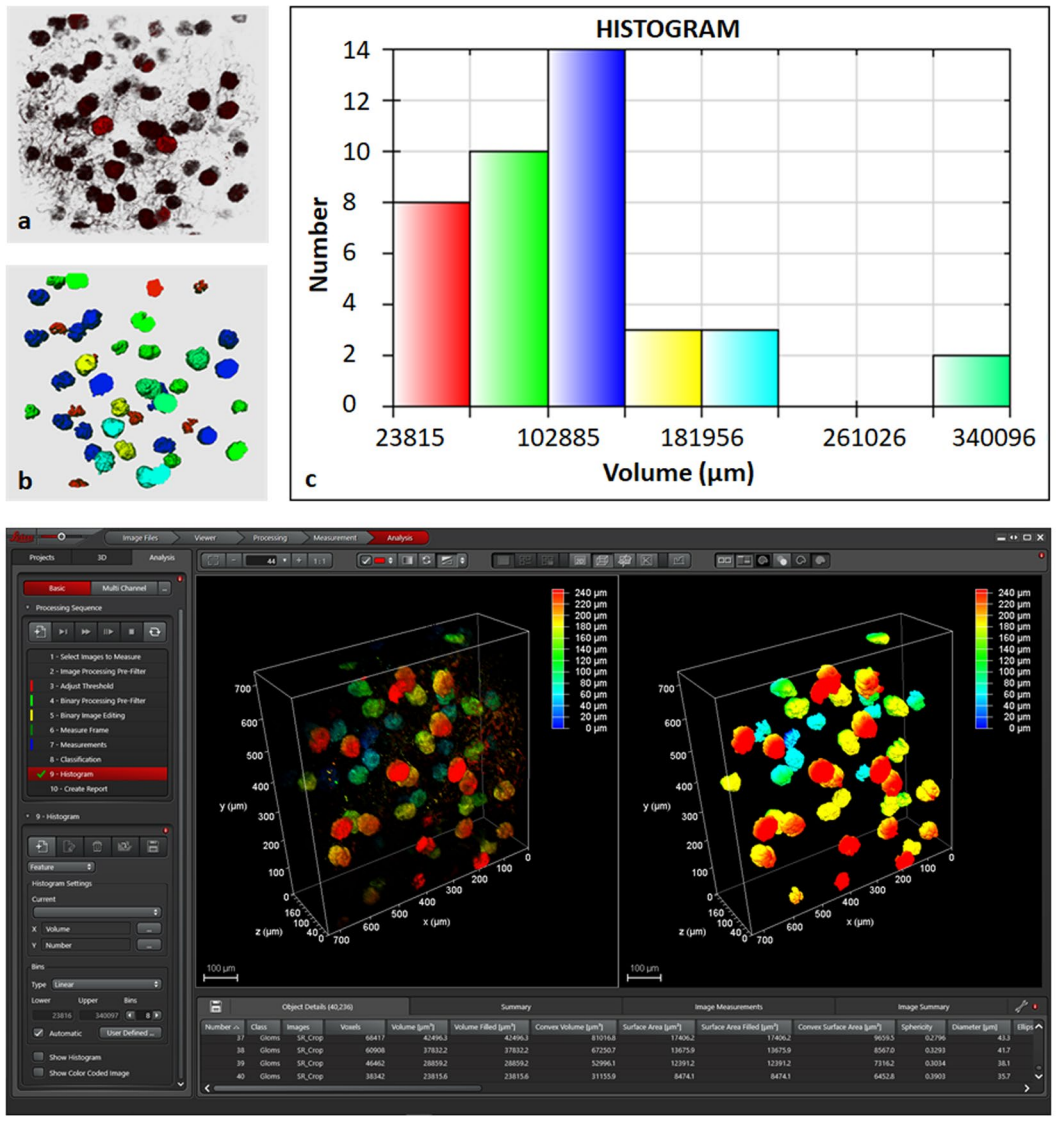
Segmentation of glomeruli from mouse kidney perfused with MHI148-PEI and cleared with ECi using simple image processing pipeline: background removal using median filter, segmentation using Otsu thresholding in combination with binary morphological filters and 2-class classifier to filter only bigger glomeruli (class 1). The partially visible glomeruli and background objects were designated class 2. (**a**) Raw 3D data from small section of left mouse kidney. The raw data with specific settings is shown in Supplementary figure S13. (**b**) Segmented 3D data showing glomeruli using different group of colors based on the volume histogram shown in (**c**). (**c**) Volume histogram showing six groups where red color represents the group with smallest. (**d**) Leica LAS X software to create custom made analysis pipeline as shown by a sequence on left. The left and right images depict raw and segmented data (only class 2).

The results of segmentation are shown in Fig. 8. In Fig. 8a, raw data of kidney containing glomeruli is shown in red shading color scheme against a gray background. The resulting segmentation using simple thresholding and morphological filters is shown in Fig. 8b. Here, only the segmented objects i.e. glomeruli are given. The color code is based on the volume histogram using six different pairs as shown in Fig. 8c.

**Figure 9:**
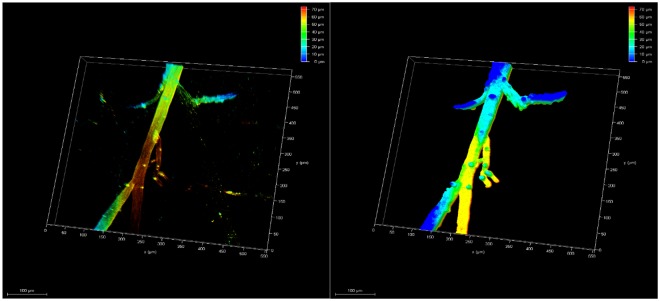
Segmentation of upper back part of mouse skin from Fig. 3b perfused with MHI148-PEI and clear with ECi using simple image processing pipeline: background removal using median filter and segmentation using Otsu thresholding in combination with binary morphological. On left: raw 3D data from small section of skin from upper back part. On right: segmentation using medial filter, thresholding and morphological filters.

The data shown is Fig. 3b from mouse skin was also used to demonstrate that simple thresholding operation can be used in such a setting to segment vessels as shown in Fig. 9.

## Discussion

In this report, we provided evidence that vascular structures in different organs as well as in glomeruli in kidneys can be visualized in a fast and highly efficient manner. We focused in this report on: (i) the synthesis of the NIR fluorescent dye MHI148-PEI; (ii) the perfusion with this dye; (iii) the rapid clearing of the organs using ECi; and (iv) the imaging of vascular structures using light microscopy. The aim of the paper was to demonstrate an efficient and shortened clearing method in combination with a cationic dye for visualizing vascular structures. The comparisons with already reported protocols were not the scope of this study. Nevertheless, it is warranted to demonstrate such comparisons in the future.

GAGs are linear polysaccharides with a repeating disaccharide unit^37^. The heterogeneity of the polysaccharides, especially the pattern of sulfation on the distal end of the chain, endows these GAGs with diverse biological functions^38^. GAGs of physiological importance are hyaluronic acid, heparin, heparan sulfate, keratan sulfate, chondroitin sulfate, and dermatan sulfate^39^. An important function of GAGs is the heparin release from granules of mast cells leading to anti-coagulation in vessels^39^. In vessel walls dermatan sulfate is the predominant antithrombotic GAGs^39^. GAGs and proteoglycan form in the glomerular basement membrane a charge-selective barrier that restricts the transmembrane flux of anionic proteins across the membrane into the urinary space^40^. We designed a cationic NIR dye based on the unique structure of vessels and the glomerular capillary wall. The key features of this dye include a high number of positive charges, NIR fluorescence emission and large molecular weight and size. The positive charges are derived from the branched PEI leading to its electrostatic attachment to GAGs and proteoglycans on the vessel walls and the glomerular basement membrane. Furthermore, the NIR spectra feature allows for a deep tissue penetration and minimizes the impact of the intrinsic auto-fluorescence background. Such a wavelength matches with almost all confocal systems, which provide an excitation laser wavelength lower than 690 nm. Importantly, both molecular weight and size of MHI148-PEI are higher than the threshold of glomerular filtration, thus preventing the filtration of the agent into the tubules and giving an extremely high signal-to-noise ratio. This can be seen by the comparison of the scans of MHI148-PEI and MHI148-PEI-S (Fig. 6a,b). To our knowledge, this is the first report of designing a NIR fluorescent agent for staining glomeruli based on its unique biological structure.

Optical tissue clearing is becoming increasingly popular. Its use, however, is severely hampered by lengthy sample processing times (the interval between organ harvesting and the start of microscopic analysis). Depending on the protocols/technologies used, this time interval may last from one day up to several weeks. In our protocol, we have reduced the processing time to only 4 hours with uncompromised preparation quality. We refined the process of organ harvesting and organ/sample staining. This was achieved by retrograde perfusion after puncturing the bifurcation of the aorta. The perfusion process was volume and pressure controlled. It consisted of saline/heparin to remove all hemoglobin followed by the staining of the organs with MHI148-PEI. The unbound MHI148-PEI was removed by a quick flush of saline/heparin. A final step with 4% PFA was performed to fix the organs. This whole process lasts about 30 min. The perfusion of the whole animal allows the harvesting of already stained organs including kidney, liver, skin, brain, muscle, pancreas, heart, and lungs.

Moreover, we optimized the subsequent dehydration and clearing processes. This involved the use of an automatic tissue processor for high throughput at room temperature and under vacuum. The process consisted of four dehydration steps lasting overall 2 hours. Finally, optical tissue clearing in ECi lasting for 2 hours was performed. ECi is an inexpensive, non-toxic substance suitable for a rapid and efficient clearing of whole organs. MHI148-PEI is compatible with the ECi tissue clearing process. The major difference to the procedure described by Klingberg *et al*.^12^ is that every processing step was performed at room temperature. Thus, the diffusion of the chemical reagents into biological tissues was accelerated and the time of the process shortened.

Cleared tissue can be imaged by using various types of microscopy including confocal and light-sheet microscopy. ECi is an organic solvent and may attack the glue of the objectives of the microscopes. As the imaging has to be performed in immersion mode, alternatives to ECi like immersion oil have successfully been tested in this study. Immersion oil has a similar refractive index (RI = 1.51) like ECi (RI = 1.558) and works perfectly well as a substitute for ECi. Thus, the risk of damaging the objectives was reduced. We observed that ECi rendered organs clear and allowed preserving the NIR fluorescence.

Klingberg *et al*. in their study have performed a shrinkage analysis of kidney after clearing. The analysis was based on the volume of the organ measured by light sheet microscopy using automated image analysis. As expected, they observed a reduction in volume and diameter of the organ. We decided to perform a shrinkage analysis not only of the kidney but also of different organs to see whether they behave in the same way. For the analysis, we calculated the weight and the area of the organs instead of the volume. The area was calculated using automated image processing.

By comparing the two approaches (manual vs. automatic), we observed a decrease in area and weight after clearing for brain, kidneys, liver and spleen, while an increase was observed for the lung. Concerning the EDL muscle, there is a significant gain in weight and a loss in area after clearing. Contrarily, the heart seems to show an opposite behavior (not statistically significant). A possible explanation could be attributed to the different nature of the organs. Indeed, different composition in macromolecules and different structure of the organs certainly play key roles in the response to the clearing protocol. This might explain why some organs are more affected by the fixation and/or dehydration steps than others.

## Conclusions

In summary, a cationic NIR dye, MHI148-PEI, was rationally designed and synthesized. It showed high hydrophilicity, favorable fluorescence properties, and deep penetration through tissue. Furthermore, we refined the process of organ harvesting and organ/sample staining by retrograde perfusion and optimized the subsequent dehydration and clearing process by the use of an automatic tissue processor for high throughput at room temperature and under vacuum. Using our approach, the time interval between organ harvesting and microscopic analysis can be reduced from day(s) or weeks to 4 hours. The ECi-based tissue clearing is also shown to be compatible with NIR dye staining. Due to the above properties, we have demonstrated that the new cationic NIR fluorescent dye in combination with commercially available confocal or light-sheet microscopes enables the visualization of vascular structures with an extremely high resolution. The application of this protocol to vascular and kidney disease models will be of utmost interest.

## Methods

### Materials and instruments

Branched PEI polyethylenimine was purchased from Polysciences Inc. Other reagents and deuterated solvents were purchased from Sigma Aldrich or Carl Roth and used as received. Silica gel (Silicycle, 230–400 mesh) was used for column chromatography. NMR spectra were recorded on a Bruker 300 MHz NMR instrument. Chemical shifts are reported in ppm relative to residual protic solvent resonances. Mestre Nova LITE v5.2.5-4119 software (Mestre lab Research S.L.) was used to analyze the NMR spectra. Matrix-assisted laser desorption/ionization time-of-flight (MALDI-TOF) analyses were collected on a Bruker ultraflex TOF/TOF instrument. UV-vis and fluorescence spectra were acquired using a microplate reader (Tecan Infinite M200). The pH of samples solution was tested by Mettler Toledo FiveEasy™ FE20pH bench meter. IUPAC names of all compounds are provided and were determined using CS ChemBioDraw Ultra 12.0. Dynamic light scattering (DLS) studies were conducted using a Malvern Zetasizer Nano S90 equipment. Images were taken by confocal microscopy and light-sheet microscopy (Leica Microsystems, Leica SP8 and Leica TCS SP8 DLS).

### Preparation and characterization of MHI148-PEI and MHI148-PEI-S

Starting from 2,3,3-trimethylindolenine (compound **1**), a carboxylic acid NIR dye (compound **4**) was synthesized by reacting the *meso* chlorine atom of MHI148 with a nucleophile 4-aminobutanoic acid. Finally, compound 4 was conjugated with branched PEI (50–100 kDa) to obtain the NIR fluorescent agent MHI148-PEI. Intermediates and products were characterized by ^1^H-NMR, ^13^C-NMR, and LR-MS (Supplementary figures).

### Synthesis of compound 2

To a 50 mL three-neck round-bottom flask, toluene (20 mL) was added to a mixture of 2,3,3-trimethylindolenine (compound **1**, 1.59 g, 10 mmol) and 6-bromohexanoic acid (1.95 g, 10 mmol). The suspension was refluxed at 110 °C for 12 hours under an inert gas atmosphere. The hot solution was cooled to room temperature and concentrated using a rotary evaporator. The residues were washed thrice (150 mL) with ether and dried to give a pink solid (compound **2**, 2.19 g, 8 mmol, 80% yield). TLC (silica gel, ethyl acetate) R_f_ = 0.3. NMR data was reported in a previous reference^41^. LR-MS (m/z): calculated: 274.18, found: 274.19.

### Synthesis of compound 3 (MHI148)

To a 50 mL three-neck round-bottom flask, a mixture of sodium salt of compound **2** (0.80 g, 2.26 mmol), Vilsmeier-Haack reagent (0.36 g, 1 mmol) and anhydrous sodium acetate (0.252 g, 3 mmol) in 20 ml of absolute ethanol was refluxed for 8 hours under argon. The reaction mixture was cooled to room temperature and then concentrated under reduced pressure to yield a brownish green residue. The crude product was washed with ethyl acetate/petroleum ether (1/2). The residues were purified by silica gel column chromatography with methanol/ethyl acetate (1/2), a dark green solid (compound **3**, MHI148, 0.557 g, 85% yield) was obtained.

^1^H NMR (300 MHz, DMSO-d_6_): δ 1.43 (m, 4 H), 1.57 (m, 4 H), δ 1.67 (s, 12 H), 1.77 (m, 4 H), 1.89 (m, 2 H), 2.21 (t, 4 H), 2.73 (t, 4 H), 4.23 (t, 4 H), 6.34 (d, J = 15, 2 H), 7.30 (m, 2 H), 7.46 (m, 4 H), 7.65(d, J = 6, 2 H) 8.28 (d, J = 12, 2 H). ^13^C NMR (300 MHz, DMSO-d_6_): δ 174.2, 172.3, 148.1, 143.0, 142.1, 141.1, 128.7, 126.2, 125.2, 122.6, 111.6, 101.7, 48.9, 43.7, 33.4, 27.4, 26.8, 25.9, 25.7, 24.1, 21.1, 20.4. LR-MS (m/z): calcd: 683.36, found: 683.32.

### Synthesis of compound 4

To a 50 mL three-neck round-bottom flask, a mixture of compound **3** (0.138 g, 0.2 mmol) and 4-aminobutanoic acid (0.062 g, 0.6 mmol) in DMSO (15 mL) was heated at 65 °C overnight under an inert gas atmosphere. The reaction mixture was cooled to room temperature and precipitated in ethyl acetate and washed with dichloromethane. The residues were purified by silica gel column chromatography and washed with methanol/ethyl acetate (1/2). A blue solid (compound **4**, 0.107 g, 71% yield) was obtained. ^1^H NMR (300 MHz, CD_3_OD): δ 1.31 (m, 4 H), 1.46 (m, 4 H), δ 1.65 (s, 12 H), 1.81 (m, 6 H), 2.05 (m, 2 H), 2.20 (m, 4 H), 2.52 (t, 2 H), 2.95 (m, 6 H), 3.92 (m, 4 H), 5.78 (d, J = 15, 2 H), 7.03 (m, 4 H), 7.34(m, 4 H) 7.74 (d, J = 15, 2 H). ^13^C NMR (300 MHz, CD_3_OD): δ 180.9, 172.1, 150.1, 144.9, 142.1, 141.2, 129.0, 123.7, 123.0, 110.0, 99.3, 41.0, 38.45, 36.4, 29.1, 27.9, 27.1, 25.0, 21.9, 20.6. LR-MS (m/z): calcd: 750.45, found: 750.46.

### Synthesis of compound MHI148-PEI

A mixture of compound 4 (0.06 g, 0.078 mmol), 1-ethyl-3-(3-dimethylaminopropyl) carbodiimide (0.06 g, 0.31 mmol), N-hydroxysuccinimide (0.041 g, 0.35 mmol) and PEI (0.60 g, 50–100 kDa) and DMSO (20 mL) was stirred at room temperature under argon gas protection and exclusion of light. After 12 hours, the reaction mixture was poured into a dialysis membrane with molecule weight cutoff 2000 Da (Spectrum Labs Inc) and two sides of dialysis tubing were tightly clamped (Supporting information, Fig. S1). The dialysis was performed thrice in PBS, a fresh PBS (2000 mL) was replaced every 12 hours and gentle stirring at room temperature was continued, the blue aqueous from the dialysis membrane was collected and was frozen dry to obtain a blue solid. Using the same procedure, MHI148-PEI, MHI148-PEI-S was produced.

### Optical properties characterization

Stock solutions of all the compounds were prepared and stored at −20 °C. All the spectroscopic measurements were conducted in PBS. UV-vis absorption and fluorescence spectra were acquired using a microplate reader (Tecan, Infinite M200). All measurements were conducted at 25 °C.

### Dynamic light scattering (DLS)

MHI148-PEI (2 mg/mL) and MHI148-PEI-S (2 mg/mL) in PBS were prepared and filtered through a sterile 0.22 μm filter before analysis. DLS studies were conducted. The Zetasizer Nano S90 uses a 633 nm helium-neon laser. The measurements were carried out in quadratic cells and the scattered light at an angle of 90° at a controlled temperature (25 °C) was analyzed. The intensity of scattering of a particle is assumed to be proportional to the sixth power of its diameter. The apparent hydrodynamic radius was calculated according to the Stokes-Einstein equation.

### Animals

Adult C57BL/6 mice were used in the study. Animals were anesthetized by an intraperitoneal injection of ketamine/xylazine (16 mg/kg BW xylazine and 120 mg/kg BW ketamine). All animal experiments were conducted in accordance with the German Animal Protection Law and approved by the local authority (Regierungspräsidium Nordbaden, Karlsruhe Germany in agreement with EU guideline 2010/63/EU).

### Overview of the protocol

#### Perfusion

**Figure 10:**
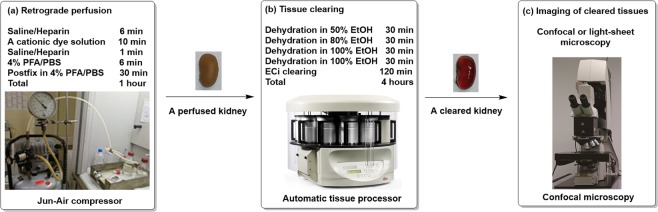
Overview of the protocol. The protocol can be divided into 3 steps: (**a**) efficient retrograde perfusion of MHI148-PEI; (b) tissue clearing including dehydration and ECi based clearing in an automatic tissue processor; and (**c**) imaging vascular structures especially vessels and glomeruli using confocal or light-sheet microscopy.

We developed a retrograde perfusion protocol by insertion of the needle into the bifurcation of the aorta, which allows the usage of all parts of an animal **(**Fig. 10, Step 1). Furthermore, the perfusion is pressure- and volume-controlled.

The perfusion device consists of a series of tubings (0.58 mm diameter) connecting a compressor (Jun-Air compressor, Rio Grande, 117301) with a set of two bottles filled with saline/heparin (0.9% saline heparin 5 IU/mL pH = 7) and 4% PFA. Additionally, a syringe pump (Chemyx, Fusion 100 infusion pump) for the application of MHI148-PEI (0.25 mg/mL in PBS) is connected to the aforementioned tubings. All tubings end in a three-way valve leading to a G25 butterfly needle. The perfusion protocol starts with flushing out the blood from the body of the animal by using saline/heparin (200 mbar, 6 min, ~40 mL), followed by staining the organs with MHI148-PEI (1 mL/min, 10 mL). To remove the excess of unbound MHI148-PEI, a short rinsing of saline/heparin (200mbar, 1 min) is performed. Lastly, fixation in 4% PFA (200mbar, 6 min, ~40 mL) is done. Organs were collected and stored in 4% PFA until processed.

#### ECi clearing protocol

The dehydration and the clearing were performed in a tissue processor (Leica microsystems, TP1020) (Fig. 10, Step 2). Organs were dehydrated by using ethanol dilution series (50%, 80%, 100%, 100%) and then cleared in ECi (Fig. 10a, Step 2). The immersion time in ethanol for each step lasts 30 min, while the ECi step lasts 2 hours, for a total of 4 hours. Immersion time in ECi can be varied and extended to overnight for large organs and tissues. Both dehydration and clearing steps were performed at room temperature to improve the diffusion of the chemical reagents into biological tissues. After clearing is performed, the organs can be stored in black tubes (Litesafe, TB5000) filled with ECi.

#### Microscopy and data acquisition

The cleared tissues were imaged either by confocal or light-sheet microscopy (Fig. 10, Step 3). The samples were positioned either on a glass slide (Menzel-Glaser, AA00008132E) or in a Petri dish (Thermo Fisher Scientific, S33580A), immersed in immersion oil (RI = 1.51, Thermo Fisher Scientific, NC0586121). Imaging was performed with a Leica SP8 confocal microscope with a HC PL APO 20x/0.75 IMM CORR CS2 objective (Leica Microsystems, 15506343). MHI148-PEI and MHI148-PEI-S were excited at 638 nm and fluorescence was detected using a Cy7 emission filter. For imaging on a light-sheet microscope (Leica Microsystems, Leica TCS SP8 DLS), samples were mounted to a hook-shaped holder, submerged in immersion oil, and evaluated with a HCX PL FLUOTAR 5x/0.15 objective. Images were processed and 3D reconstruction was rendered using Leica LAS X software. All the data acquired were saved as. lif files and read into LAS X. The images from 3D scan data contained in this article are based on the adjustments made in minimum and maximum intensity, shading, gamma and opacity of 3D viewer section in the LAS X software.

### Image analysis

To demonstrate the beneficial effect of using MHI148-PEI and ECi clearing on samples, we used kidney samples as an example for quantification. The goal was to see if simple automatic image processing methods can easily segment and quantify the structures in a small section of a kidney. For simplicity, we developed an image analysis pipeline in Leica LAS X software using median filter for noise removal, thresholding in combination with morphological filters for image segmentation and simple two-class classification to differentiate between glomeruli and background objects e.g. tubuli, noise etc. Three glomeruli were labeled manually as class 1 for supervision of the machine learning classifier. The manual count (n = 43) was also done using the same software and quantitative analysis showed 40 class 1 objects were found. The error is due to the fact that labeling was done using completely visible glomeruli only. However, in manual counting even partially detected glomeruli were considered. Moreover, the software is able to extract different shape and size features for the objects detected. The average volume of glomeruli was found to be 206594 cubic microns.

### Shrinkage analysis

After perfusion, the following organs were collected: right and left kidney, left lung, heart, left lateral lobe of the liver, brain, EDL muscle. 2D images of organs were taken before and after clearing using iPhone 6 s camera with flash ON. An additional scale (1 cm) visible in each acquired image was inserted. The automatic image processing pipeline employs:

- median filtering 3 × 3 on the images,
- segmentation based on fixed threshold,
- morphological filtering operations to obtain the biggest binary object. The morphological operations consisted of opening, hole filling (1), dilation and hole filling (2), and
- particle filtering (using 2-class problem)

Similarly, the number of pixels were counted for the scale and each pixel was defined (in cm) based on this scale. Using this pixel definition, number of pixels were calculated in the segmented region and multiplied with the scale to get the area in cm square. The total area of an organ before clearing was taken as 100%. The area calculated after the clearing procedure was normalized in each individual case with respect to total area before clearing. The resulting values are displayed in bar plot shown in Fig. 2. Because of their different nature, structure and composition, organs were weighed before and after clearing in order to analyze prospective changes in weight due to their response to the clearing protocol.

The result is displayed in bar plot shown in Fig. 2.

### Statistical analysis

To measure the shrinkage of different organs before and after clearing, each organ was measured twice, resulting in pairs of observations. Therefore, a paired t-test was chosen as the most suitable test. A p-value < 0.05 was considered as statistically significant. All statistical calculations were performed using SAS JMP 13.0.0 (SAS Institute, Cary, NC, USA).

## Electronic supplementary material

Supplementary information

## Data Availability

The authors declare that all relevant data supporting the findings of this study are available within the article and in the Supplementary Information document, or from the corresponding author on request.

## References

[1] Hong G (2012). Multifunctional *in vivo* vascular imaging using near-infrared II fluorescence. Nat Med.

[2] Hoy WE (2008). Nephron number, glomerular volume, renal disease and hypertension. Curr Opin Nephrol Hypertens.

[3] Beeman SC (2011). Measuring glomerular number and size in perfused kidneys using MRI. Am J Physiol Renal Physiol.

[4] Brenner BM, Garcia DL, Anderson S (1988). Glomeruli and blood pressure. Less of one, more the other?. Am J Hypertens.

[5] Heilmann M (2012). Quantification of glomerular number and size distribution in normal rat kidneys using magnetic resonance imaging. Nephrol Dial Transplant.

[6] Liu X (2017). Hemodynamics analysis of the serial stenotic coronary arteries. BioMed Eng OnLine.

[7] Yang Y (2017). Impact of spatial characteristics in the left stenotic coronary artery on the hemodynamics and visualization of 3D replica models. Sci Rep.

[8] Maruyama H, Yabu Y, Yoshida A, Nawa Y, Ohta N (2000). A role of mast cell glycosaminoglycans for the immunological expulsion of intestinal nematode, Strongyloides venezuelensis. J Immunol.

[9] Baldelomar EJ (2016). Phenotyping by magnetic resonance imaging nondestructively measures glomerular number and volume distribution in mice with and without nephron reduction. Kidney Int.

[10] Wright AT, Zhong Z, Anslyn EV (2005). A functional assay for heparin in serum using a designed synthetic receptor. Angew Chem Int Ed Engl.

[11] Wang W (2015). Supercharged fluorescent protein as a versatile probe for the detection of glycosaminoglycans *in vitro* and *in vivo*. Anal Chem.

[12] Klingberg A (2017). Fully Automated Evaluation of Total Glomerular Number and Capillary Tuft Size in Nephritic Kidneys Using Lightsheet Microscopy. J Am Soc Nephrol.

[13] Richardson DS, Lichtman JW (2015). Clarifying Tissue Clearing. Cell.

[14] Bernier-Latmani J, Petrova TV (2016). High-resolution 3D analysis of mouse small-intestinal stroma. Nat Protoc.

[15] Susaki EA (2014). Whole-brain imaging with single-cell resolution using chemical cocktails and computational analysis. Cell.

[16] Spalteholz, W. über das Durchsichtigmachen von menschlichen und tierischen Präparaten. *S*. *Hirzel* (1914).

[17] Yu T (2017). Elevated-temperature-induced acceleration of PACT clearing process of mouse brain tissue. Sci Rep.

[18] Fretaud M (2017). High-resolution 3D imaging of whole organ after clearing: taking a new look at the zebrafish testis. Sci Rep.

[19] Neckel PH, Mattheus U, Hirt B, Just L, Mack AF (2016). Large-scale tissue clearing (PACT): Technical evaluation and new perspectives in immunofluorescence, histology, and ultrastructure. Sci Rep.

[20] Sung K (2016). Simplified three-dimensional tissue clearing and incorporation of colorimetric phenotyping. Sci Rep.

[21] Hama H (2011). Scale: a chemical approach for fluorescence imaging and reconstruction of transparent mouse brain. Nat Neurosci.

[22] Susaki EA (2015). Advanced CUBIC protocols for whole-brain and whole-body clearing and imaging. Nat Protoc.

[23] Erturk A (2012). Three-dimensional imaging of solvent-cleared organs using 3DISCO. Nat Protoc.

[24] Renier N (2014). iDISCO: a simple, rapid method to immunolabel large tissue samples for volume imaging. Cell.

[25] Ke MT, Fujimoto S, Imai T (2013). SeeDB: a simple and morphology-preserving optical clearing agent for neuronal circuit reconstruction. Nat Neurosci.

[26] Kuwajima T (2013). ClearT: a detergent- and solvent-free clearing method for neuronal and non-neuronal tissue. Development.

[27] Perbellini. F (2017). Free-of-Acrylamide SDS-based Tissue Clearing (FASTClear) for three dimensional visualization of myocardial tissue. Sci Rep.

[28] Lee E (2016). ACT-PRESTO: Rapid and consistent tissue clearing and labeling method for 3-dimensional (3D) imaging. Sci Rep.

[29] Aoyagi Y, Kawakami R, Osanai H, Hibi T, Nemoto T (2015). A rapid optical clearing protocol using 2,2′-thiodiethanol for microscopic observation of fixed mouse brain. PLoS One.

[30] Dodt HU (2007). Ultramicroscopy: three-dimensional visualization of neuronal networks in the whole mouse brain. Nat Methods.

[31] Erturk A (2011). Three-dimensional imaging of the unsectioned adult spinal cord to assess axon regeneration and glial responses after injury. Nat Med.

[32] Longmire M, Choyke PL, Kobayashi H (2008). Clearance properties of nano-sized particles and molecules as imaging agents: considerations and caveats. Nanomedicine.

[33] Ruggiero A (2010). Paradoxical glomerular filtration of carbon nanotubes. Proc Natl Acad Sci USA.

[34] Wu H, Huang J (2016). PEGylated Peptide-Based Imaging Agents for Targeted Molecular Imaging. Curr Protein Pept Sci.

[35] Wang Y, Zhang DH, Zhang JY, Chen N, Zhi GY (2016). High-yield synthesis of bioactive ethyl cinnamate by enzymatic esterification of cinnamic acid. Food Chem.

[36] Papakonstantinou E, Karakiulakis G (2009). The ‘sweet’ and ‘bitter’ involvement of glycosaminoglycans in lung diseases: pharmacotherapeutic relevance. Br J Pharmacol.

[37] Beahm BJ (2014). A visualizable chain-terminating inhibitor of glycosaminoglycan biosynthesis in developing zebrafish. Angew Chem Int Ed Engl.

[38] Mende M (2016). Chemical Synthesis of Glycosaminoglycans. Chem Rev.

[39] Oohira A, Wight TN, Bornstein P (1983). Sulfated proteoglycans synthesized by vascular endothelial cells in culture. J Biol Chem.

[40] McCarthy KJ, Wassenhove-McCarthy DJ (2012). The glomerular basement membrane as a model system to study the bioactivity of heparan sulfate glycosaminoglycans. Microsc Microanal.

[41] Yeh C (2013). Tumor targeting and MR imaging with lipophilic cyanine-mediated near-infrared responsive porous Gd silicate nanoparticles. Biomaterials.

