# Supplementary material for: A cationic near infrared fluorescent agent and ethyl-cinnamate tissue clearing protocol for vascular staining and imaging
Source: Sci Rep. 2019 Jan 24;9:521. doi: 10.1038/s41598-018-36741-1 (PMC6345820; doi:10.1038/s41598-018-36741-1)
Supplement: Supplementary file 1 — Supplementary information [file 41598_2018_36741_MOESM1_ESM.docx]

***Supporting information***

**A cationic near infrared fluorescent agent and ethyl-cinnamate tissue clearing protocol for vascular staining and imaging**

Jiaguo Huang^1,2^, Cinzia Brenna^1,2^, Arif ul Maula Khan^1,2^, Cristina Daniele^1,2^, Rüdiger Rudolf ^2,3^, Vincent Heuveline^4^, and Norbert Gretz^1,2*^

^1^Medical Research Center, Medical Faculty Mannheim, University of Heidelberg, Theodor-Kutzer-Ufer 1-3, 68167, Mannheim, Germany

^2^Institute for Medical Technology, University of Heidelberg and University of Applied Sciences, Theodor-Kutzer-Ufer 1-3, 68167, Mannheim, Germany

^3^Institute of Molecular and Cell Biology, Mannheim University of Applied Sciences, 68163, Mannheim, Germany

^4^Director of the Computing Centre, Heidelberg University, Im Neuenheimer Feld 293, 69120 Heidelberg, Germany

Correspondence should be addressed to N.G. (Norbert.Gretz@medma.uni-heidelberg.de)


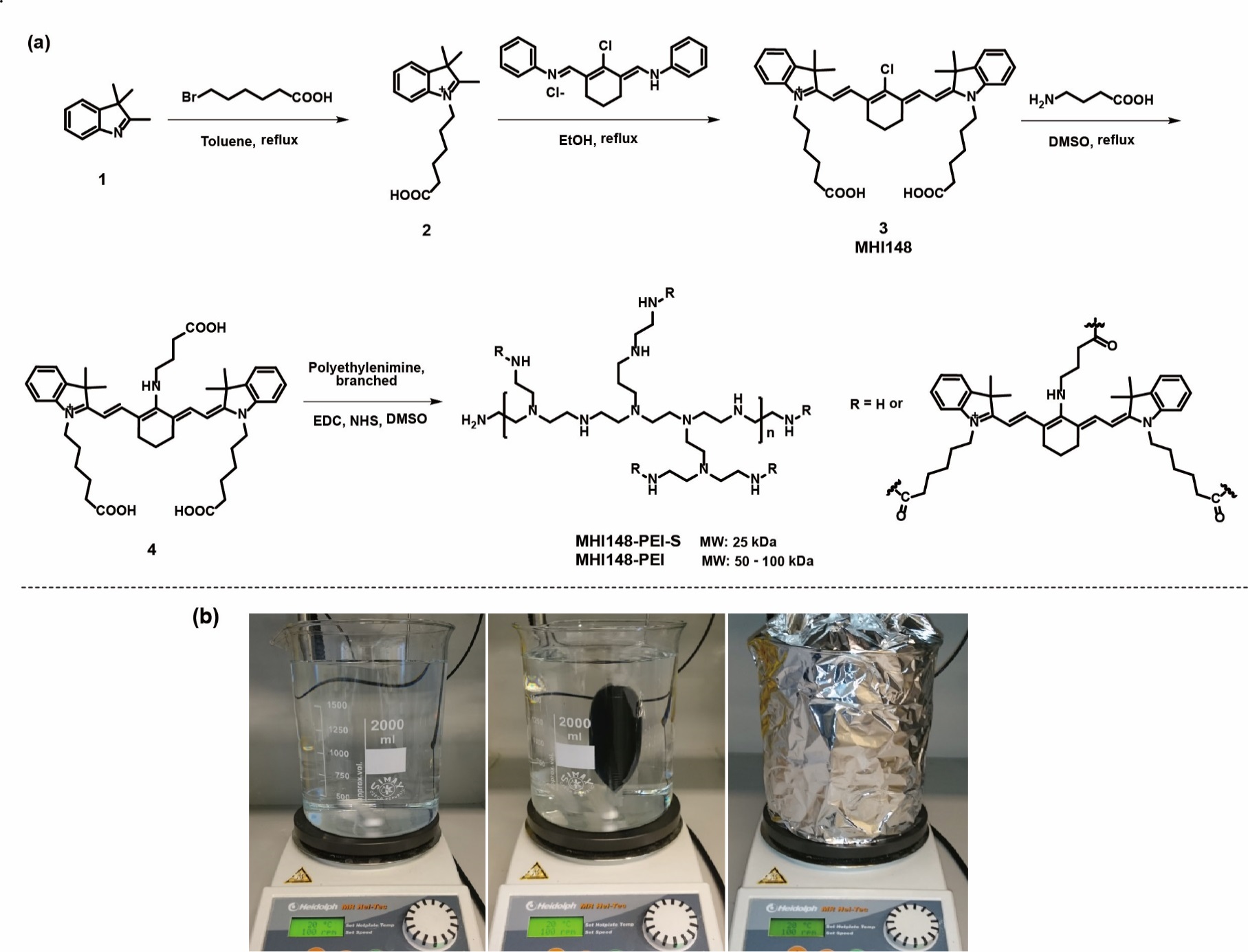


**Figure S1.** Pour the crude product (MHI148-PEI or MHI148-PEI-S) into a dialysis membrane with molecule weight cutoff 2000 Da, and clamp two sides of dialysis tubing tightly. Perform the dialysis thrice by against with PBS (1x), replace a fresh PBS (2000 mL) each 12 hours and continue gentle stirring at room temperature, collect the blue aqueous from the dialysis membrane and freeze dry to obtain a blue solid.

(a)

(b)

(c)

**Figure S2.** Absorption and emission spectra of each of the dyes in PBS. (a) Compound 3 (MHI148); (b) Compound 4; (c) MHI148-PEI-S.


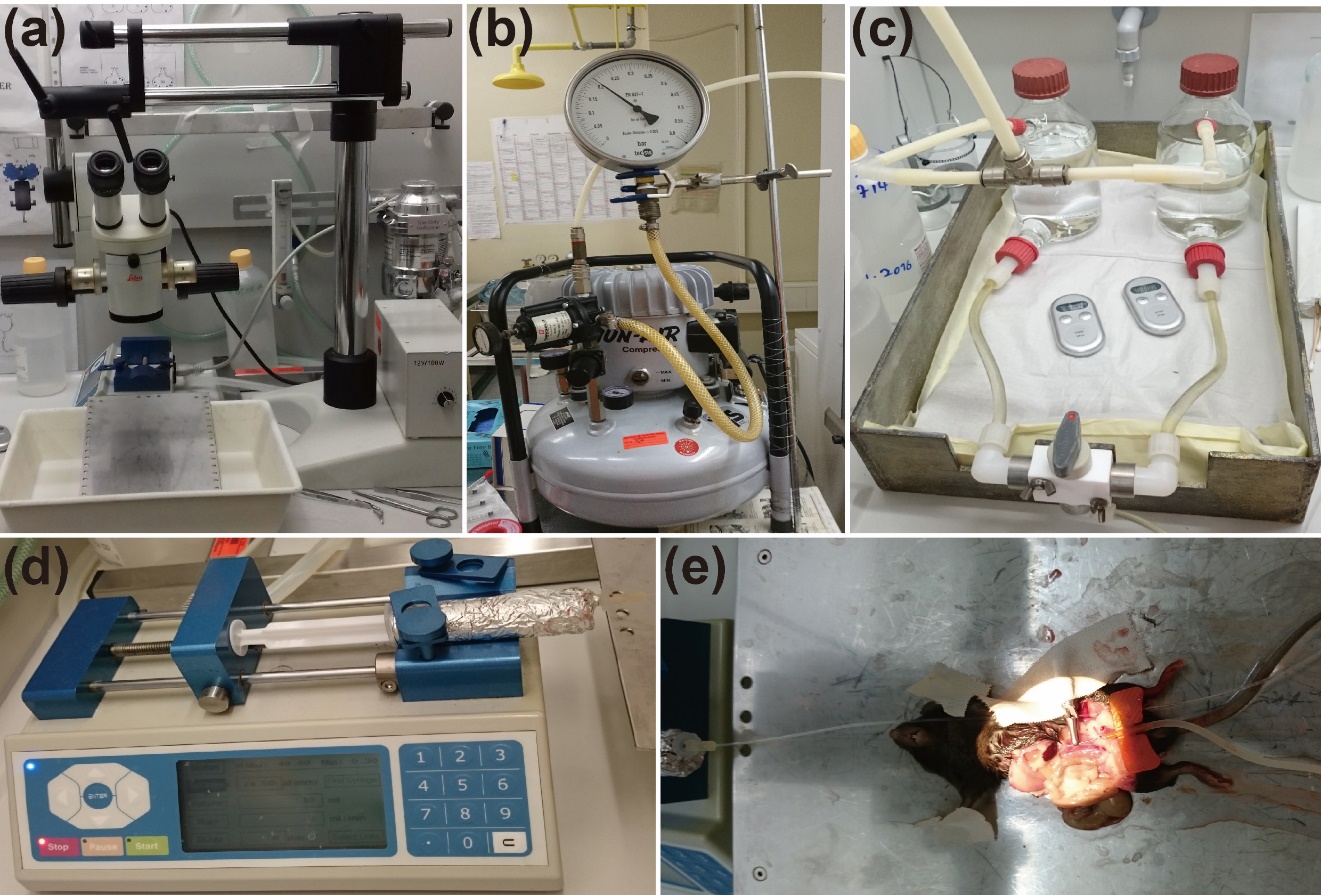


**Figure S3.** Procedure of perfusion in mouse. (**a**) Set up surgery tools for ease of access, a surgical plane is fixed and under a surgical microscope. (**b**) A Jun-Air compressor is connected with the containers and the pressure is fixed. (**c**) Heparin*/*saline and 4% PFA/PBS (wt/vol) are filled into two separated containers. (**d**) A 10 mL syringe with solution of dye MHI148-PEI and attach to fixative tubing are fixed on the infusion syringe pump system. (**e**) A mouse is under perfusion after anesthesia and surgical stages. All experiments were conducted in accordance with the German Animal Protection Law and approved by the local authority.

**Table S1** perfusion procedures in mice

| Substances | Time (min) | Pressure (mBar) |
| --- | --- | --- |
| Saline/Heparin | 6 | 200-300 |
| MHI148-PEI in PBS | 10 | 200-300 |
| Saline/Heparin | 1 | 200-300 |
| 4% PFA/PBS | 6 | 200-300 |

*
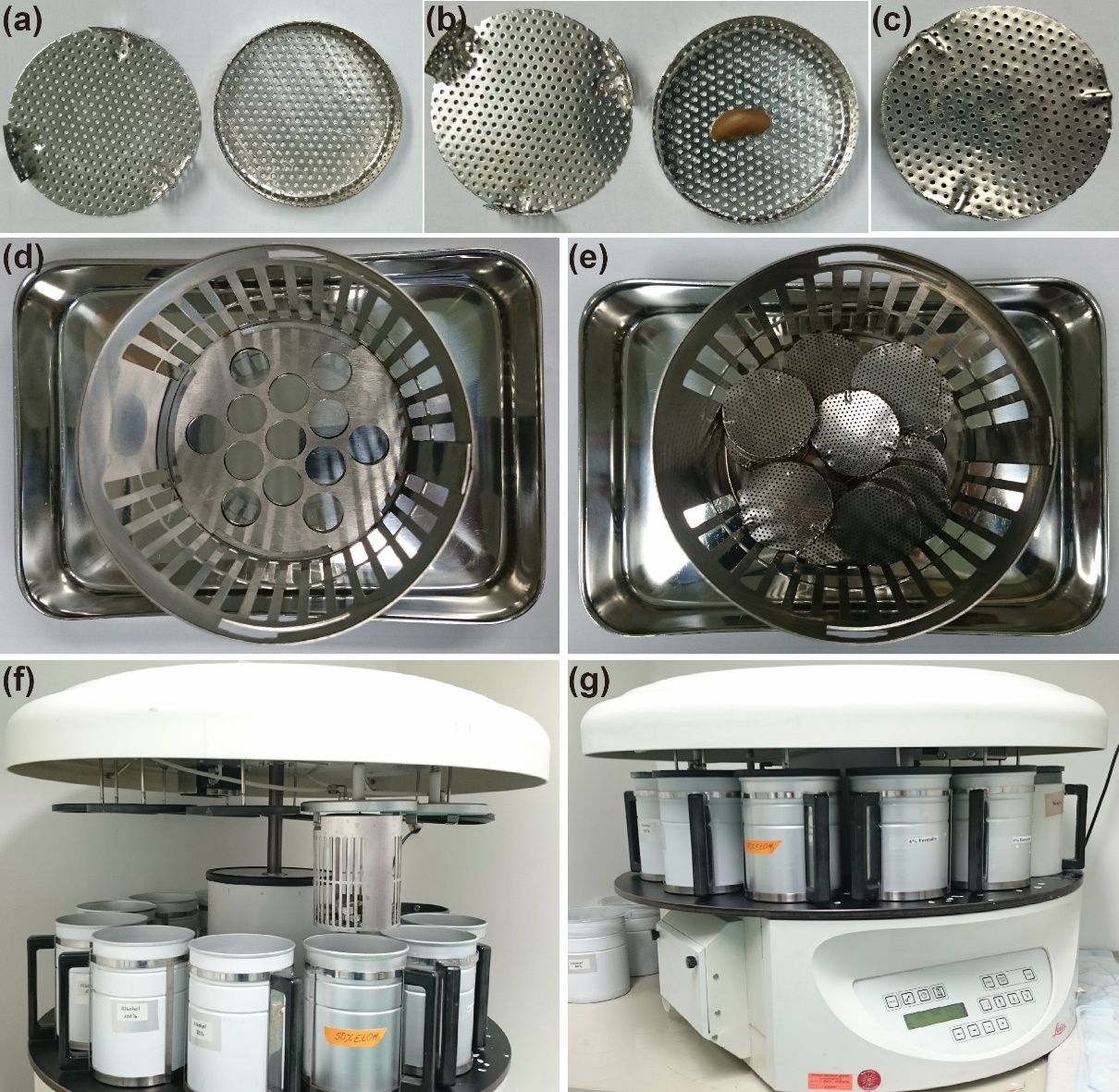
*

**Figure S4.** Clearing steps based on automatic tissue processor. (**a**) A cassette for organs holding. (**b**) An entire kidney was put into a cassette. (**c**) A cassette was fixed with an entire kidney inside. (**d**) A basket for cassette holding. (**e**) Cassettes with different organs were put into a basket. (**f**) A basket was fixed on the automatic tissue processor, which has different containers with 50%, 80%, twice 100% of EtOH and ECi. (**g**) Start to run the automatic tissue processor after a programmable setting.

**Table S2** Tissue clearing procedures

| Procedure | solvent | Time | Temperature |
| --- | --- | --- | --- |
| Dehydration | 50% EtOH | 30 min | 21°C |
|  | 80% EtOH | 30 min |  |
|  | 100% EtOH | 30 min |  |
|  | 100% EtOH | 30 min |  |
| Clearing | ECi | 2 hours | 21°C |


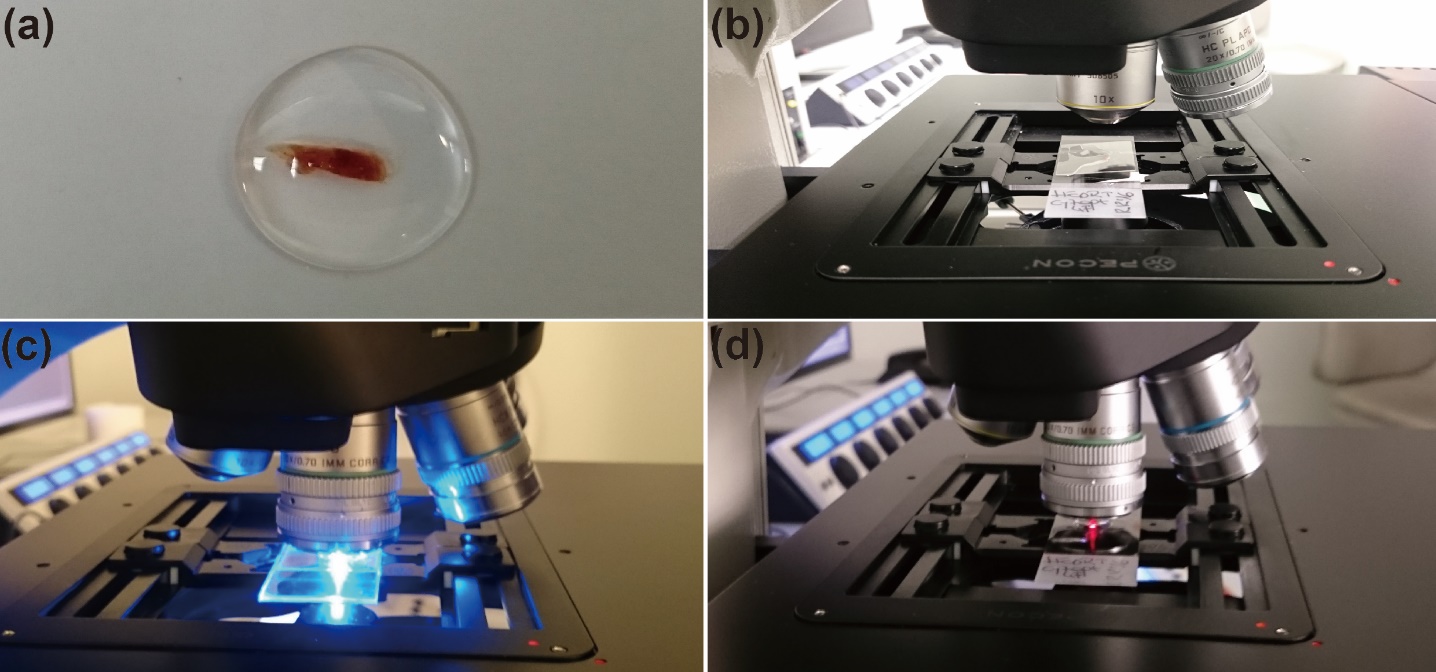


**Figure S5.** Imaging of cleared tissue using confocal microscope. (**a**) A section of cleared tissue is immersed into immersion oil (RI = 1.51) and mounted on a glass slide. (**b**) Place the slide with the tissue section under the confocal microscopy and check the focus by 10x objective. (**c**) Check the focus by 20x objective and set all the parameters before start the scan. (**d**) Start the scan using 20x objective.

**

**Figure S6.** Mass spectra of Compound 2.

*
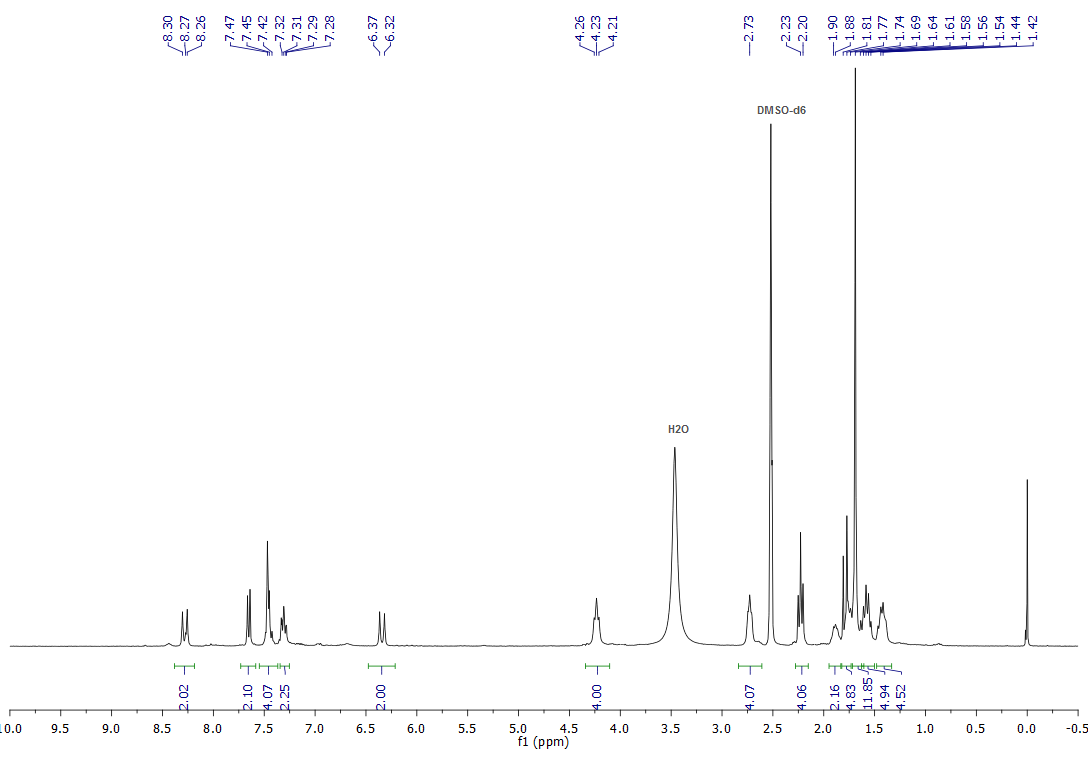
*

**Figure S7.** ^1^H-NMR of Compound 3 in DMSO-d_6_**.**

**
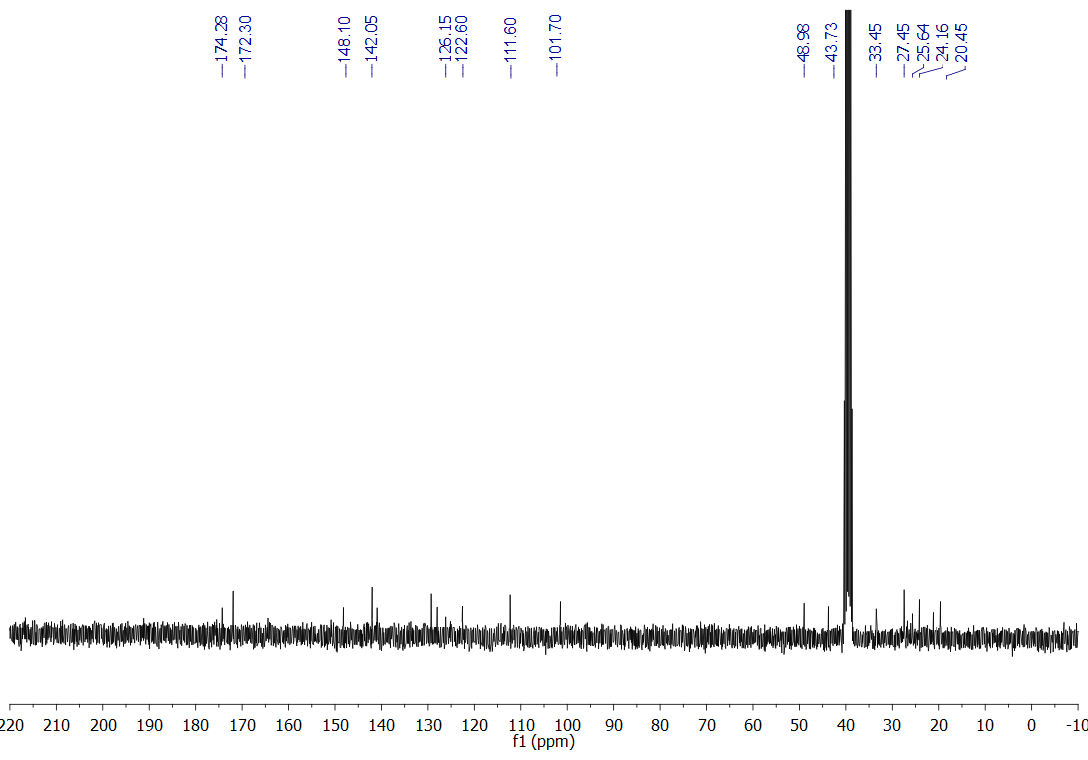
**

**Figure S8.** ^13^C-NMR of Compound 3 in DMSO-d_6._

******

**Figure S9.** Mass spectra of Compound 3.

*
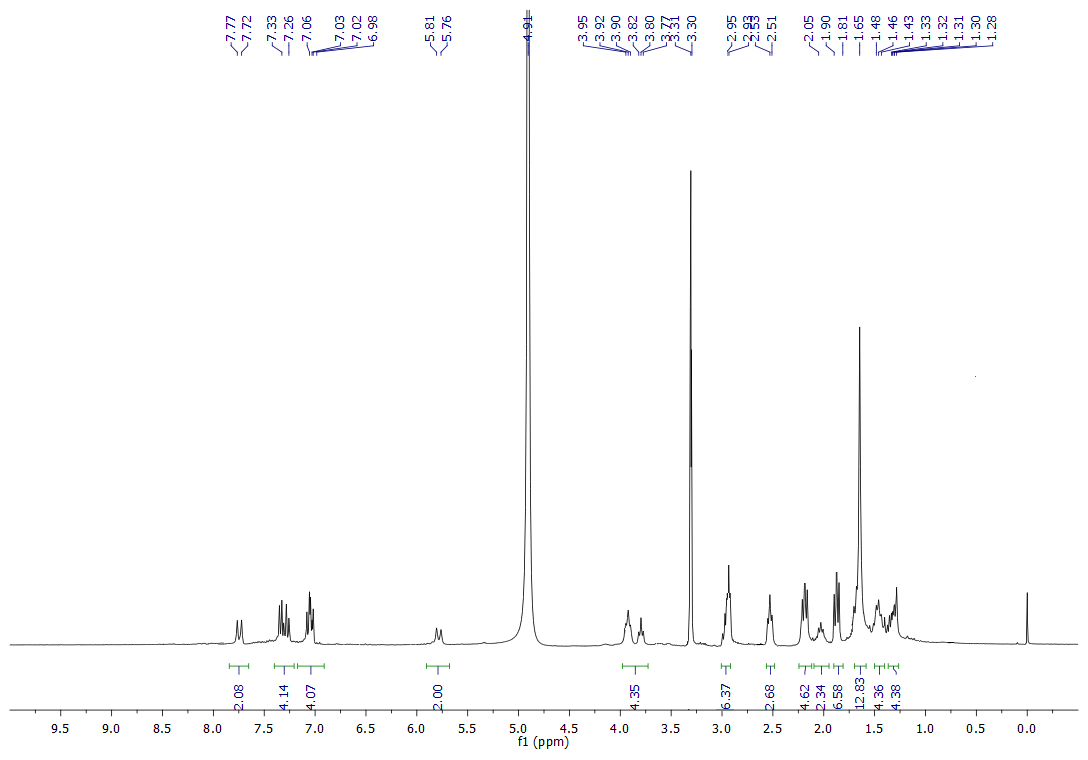
*

**Figure S10.** ^1^H NMR of Compound 4 in CD_3_OD.

**
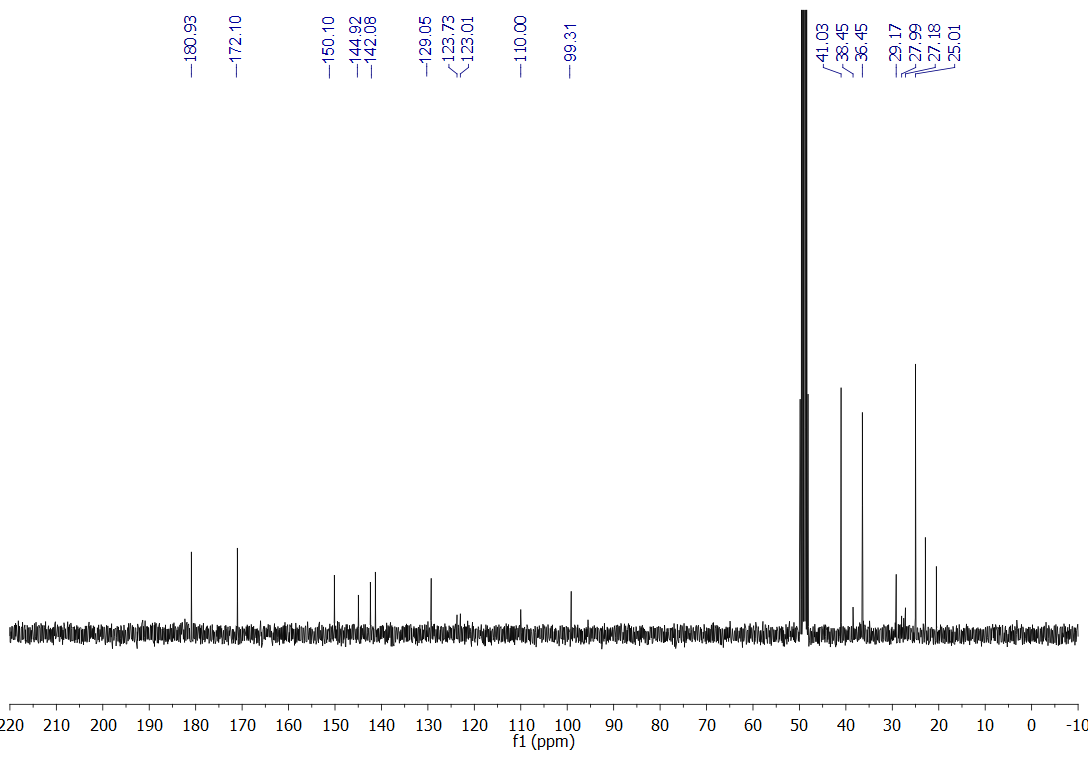
**

**Figure S11.** ^13^C NMR of Compound 4 in CD_3_OD.

**

**Figure S12.** Mass spectra of Compound 4.

*
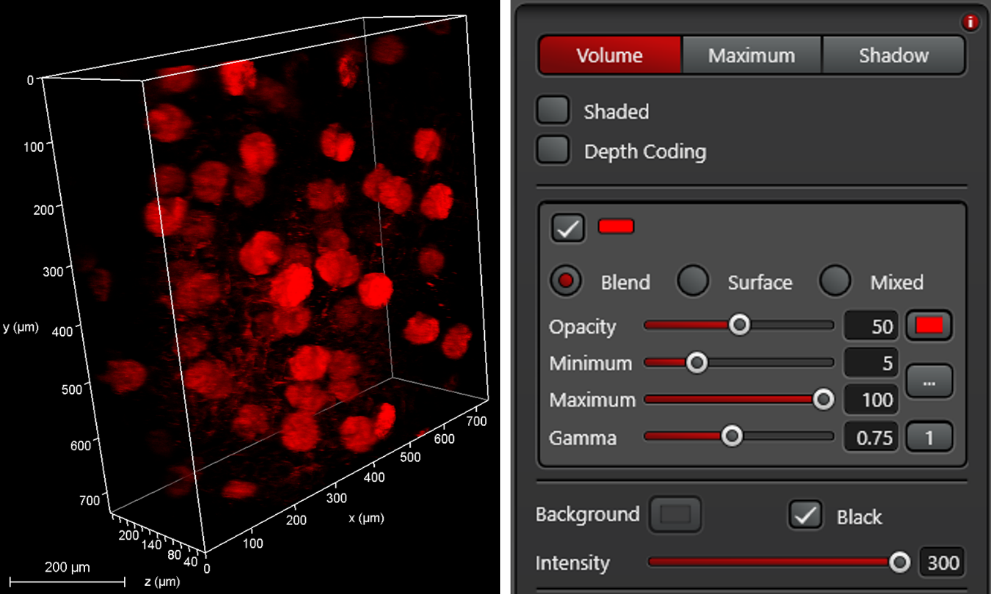
*

**Figure S13.** Raw data as displayed in LASX software for glomerular counting and sizing. The parameters used to display the raw data (on left) are shown on right.
